# Heat-health warning systems for outdoor workers: recent advances, implementation gaps, and future priorities

**DOI:** 10.3389/fpubh.2026.1925435

**Published:** 2026-08-18

**Authors:** Junjie Wei

**Affiliations:** School of Intelligent Engineering, Shandong Management University, Jinan, China

**Keywords:** heat action plans, heat-health warning systems, implementation fidelity, occupational heat stress, outdoor workers, wet-bulb globe temperature

## Abstract

Heat-health warning systems (HHWSs) are moving from population-level alerts toward occupationally relevant forecasts, task-adjusted thresholds, digital guidance, and warning-linked workplace controls. Outdoor workers nevertheless remain under-protected because regional forecasts may not represent job-level exposure and because alert receipt does not ensure that work is modified. This Mini Review critically synthesizes evidence across an occupational warning-to-action pathway: hazard forecasting, task-level risk translation, warning reach and comprehension, workplace action, and health, safety, and equity outcomes. A structured narrative search and source audit were conducted using literature available through July 2026. All cited sources were characterized by design, occupational directness, outcome proximity, implementation reporting, major bias concerns, transferability, and confidence for the specific claim supported. Recent progress includes WBGT forecasting, task and clothing adjustments, on-site verification, physiological prediction, internet of things (IoT)-enabled early warning, and warning-linked multifaceted interventions. Confidence is higher for the occupational heat burden and moderate for field action packages that reduce physiological strain or dehydration; evidence for policy-linked injury reductions is low-to-moderate because of residual confounding, and evidence that digital personalization reduces illness or injury remains low. Key controversies concern standardized vs. adjusted thresholds, advisory vs. mandatory controls, and digital personalization vs. validation, privacy, surveillance, and unequal access. The preventive value of occupational HHWSs therefore depends not only on forecast accuracy but also on decision authority, resources, enforcement, wage protection, worker trust, and the capacity to act.

## 1 Introduction

Climate change is increasing the frequency, duration, and intensity of hazardous heat, while essential outdoor work continues in agriculture, construction, transport, utilities, waste services, emergency response, and other sectors. The 2025 World Health Organization-World Meteorological Organization guidance identifies occupational heat stress as a growing health and productivity threat and calls for heat action plans adapted to industries, regions, and

worker populations (1). The International Labor Organization similarly emphasizes that climate and labor policies must be translated into effective workplace prevention (2). Outdoor heat exposure is associated with symptoms, reduced work capacity, productivity loss, kidney and cardiovascular effects, and occupational injuries, although risks vary by climate, workload, clothing, acclimatization, and worker characteristics (3–7).

HHWSs combine weather or biometeorological forecasts, risk thresholds, communication, and pre-specified responses. For this review, a complete occupational HHWS is defined as a system linking forecast or monitoring information to a threshold, communication to workplace decision-makers or workers, and an occupational response protocol. Studies evaluating only forecasting, sensing, personal cooling, or workplace controls are treated as component studies rather than complete HHWS evaluations. This distinction is important because technical performance at one stage does not establish that the full system prevents illness or injury.

Existing syntheses have examined population-level HHWS design and health outcomes, occupational heat hazards and prevention recommendations, or broader adaptation and policy responses among outdoor workers (8–11). Less attention has been given to how occupational forecasting, task-level translation, organizational authority, implementation fidelity, and worker equity interact across the warning-to-action pathway. This Mini Review therefore aims to critically synthesize recent evidence across that pathway, compare the evidence supporting standardized vs. adjusted thresholds and advisory vs. mandatory controls, and identify the system components, implementation conditions, and worker groups for which protection remains uncertain. Its contribution is to distinguish complete systems from component evidence and to make the intermediate steps between an alert and a health outcome analytically visible.

## 2 Review approach

A structured, purposive narrative search and source audit were conducted, covering literature available through July 2026. PubMed was searched using combinations of heat-health warning system, heat alert, occupational heat stress, wet-bulb globe temperature, heat action plan, outdoor work, agriculture, construction, migrant worker, wearable, and implementation. Google Scholar, publisher searches, DOI verification, and backward citation searching supplemented discovery and verification. English-language literature published from January 2019 onward was prioritized to identify recent developments; earlier sources were retained when they provided foundational guidance, standards, system descriptions, or influential field interventions. Search procedures and the selection audit are provided in Supplementary Table S1.

The final source set comprised 43 cited publications. Each source is traceable in Supplementary Table S2 by selection route, synthesis role, and retention rationale. Because the review used a structured narrative design rather than a prospectively registered systematic-review protocol, record-level database yields and PRISMA-style exclusion counts are not reported.

Eligible sources addressed at least one pathway component: hazard forecasting, task-level risk translation, warning reach or comprehension, workplace action, or health, safety, productivity, cost, and equity outcomes. General-population warning studies without occupational relevance and technologies unrelated to heat-risk assessment or protective response were not treated as core evidence. Each source received a claim-specific appraisal of design, occupational directness, outcome proximity, implementation reporting, bias concerns, and transferability. Confidence was described as higher, moderate, low-to-moderate, low, or contextual/not rated for the particular claim supported; these narrative judgments are not formal GRADE ratings. Findings were synthesized narratively, and causal language was reserved for designs capable of supporting it.

## 3 Recent advances

### 3.1 Occupationally relevant forecasting and exposure assessment

Recent systems increasingly move beyond city-level air temperature by using occupationally relevant indicators and finer spatial and temporal resolution. HEAT-SHIELD demonstrated a European platform that forecasts wet-bulb globe temperature (WBGT) and links heat risk with work intensity, clothing, and acclimatization (12). WORKLIMATE extended this approach in Italy through limited-area weather models intended to support worker health and productivity (13). In California agriculture, high-resolution forecasts were converted into WBGT-based rest-break estimates that varied by location, season, shift, and acclimatization status (14). These studies show that regional forecasts can be translated toward worksite and task decisions, but they primarily establish modeling or forecast utility rather than downstream health effectiveness.

Advance forecasts do not eliminate the need for workplace measurement. Standard weather stations may miss direct sun, radiant heat, restricted airflow, equipment-generated heat, and mobile work patterns. WBGT is a screening metric rather than a direct measure of individual strain and depends on credible estimates of metabolic rate and clothing adjustment. Guidance therefore supports combining forecasts with on-site verification and conservative interpretation for new workers, heavy work, or impermeable personal protective equipment (1, 15). Multi-day forecasts may also identify cumulative strain because some heat-exposed workers remain physiologically stressed after a shift and may not fully recover before the next exposure (16).

Recent construction studies add finer spatial and physiological layers. A 2026 analysis mapped hourly heat stress and thermal discomfort across construction locations and heights, identifying peak exposure periods and spatial hotspots relevant to scheduling and regulation (17). A modeling study predicted hourly core temperature, skin temperature, and heart rate under extreme heat scenarios and proposed integration with decision-support platforms (18). Both extend task-level translation, but neither demonstrates that alerts changed work or reduced illness.

### 3.2 Task-specific and digital risk translation

Task-level risk translation is defined here as conversion of regional or worksite heat information into decisions that account for workload, solar exposure, clothing or PPE, acclimatization, exposure duration, and changing tasks. ClimApp combines weather information with activity and personal characteristics to provide individualized thermal guidance, and usability research indicates that clarity and practical relevance influence adoption (19, 20). Wearable research has examined continuous environmental or physiological monitoring and real-time feedback for heat-exposed outdoor workers (21). An IoT-based construction system further combined portable weather stations with smart vests and generated hierarchical early warnings from environmental and worker-temperature data (22). These studies support technical and usability feasibility more clearly than reductions in heat illness or injury.

Risk translation is dynamic rather than a one-time classification. Work intensity, solar exposure, PPE, and recovery opportunities can change within a shift, while physiological signals may vary for reasons unrelated to heat. Transparent task matrices are useful for collective crew decisions; individual monitoring may provide supplementary escalation or clinical follow-up. Systems should state which decision each layer supports, how false alarms and missing data are handled, and whether worker-level data can influence employment or productivity management.

### 3.3 Integration with workplace heat action plans

Warning information has greater preventive potential when linked to pre-specified actions. Within an occupational HHWS, water, shade, acclimatization, work-rest arrangements, task rescheduling, mechanical assistance, buddy monitoring, and emergency procedures are response components rather than stand-alone warning systems (23–25). Field interventions among sugarcane workers indicate that combined water, rest, shade, work redesign, and organizational support can reduce heat strain or dehydration without necessarily reducing productivity (26–28). These intervention studies provide direct evidence for action packages, but they generally did not test a complete forecast-triggered HHWS.

A 2025 cluster randomized trial among 528 outdoor power-grid workers in southern China evaluated a multifaceted programme combining app-based education, personal protective equipment, heat-illness risk monitoring, and posters (29). Heat-related illness incidence was 1.80% in the intervention group and 4.82% in the control group by the final follow-up. The adjusted odds ratio was statistically significant at the second follow-up (0.38, 95% CI 0.15–0.97), whereas later estimates were imprecise. This trial provides moderate direct evidence for a multi-component prevention programme, but it does not isolate the contribution of the monitoring or warning component.

Policy-linked evaluations provide more direct, although context-specific, evidence. Temporary bans on outdoor work during WORKLIMATE HIGH-risk periods were associated with lower construction injury rates and a non-significant reduction in agricultural injuries in Italy (30). In Guangzhou, a labor-protection policy was associated with reductions in heat-attributable occupational injuries and insurance costs (31). These findings provide preliminary support for stronger organizational triggers, but effects cannot be separated fully from enforcement, employer capacity, wage arrangements, and concurrent policy changes. Comparative evidence on complete alert-linked action packages remains limited.

Heat action plans also clarify decision authority. Pre-season planning addresses equipment, staffing, training, acclimatization, and emergency readiness; shift-level decisions address work modification and escalation. On multi-employer sites, responsibility may be divided among principal contractors, subcontractors, supervisors, and occupational health staff. Few studies report decision timelines, actual uptake, or how responsibility disputes are resolved (23, 32). Implementation fidelity is therefore defined as the extent, timing, and consistency with which prescribed actions are delivered after a warning.

### 3.4 Communication and equity-oriented implementation

Implementation research increasingly examines whether workers receive, understand, and can act on alerts. Migrant and ethnic-minority outdoor workers may face language barriers, limited work control, piece-rate incentives, insecure contracts, and fear of job loss. A 2025 scoping review found that changing hours or activities was less common than drinking water, while qualitative studies described economic pressures to work faster or take fewer breaks (32). Reviews and participatory research also identify barriers involving shade, compensation, work culture, training, and speaking up (33, 34).

Recent recommendations favor multilingual and redundant communication, trusted intermediaries, and stronger links between alerts and rural or occupational health infrastructure (35). Evidence from the Middle East and North Africa shows that many policies still rely on calendar-based midday bans or isolated administrative measures, whereas fewer settings combine heat monitoring, training, environmental alerts, and layered controls (36). Broader reviews identify limited intervention uptake in low- and middle-income and informal settings despite high exposure (37, 38). Equity outcomes are defined here as differences between worker groups in warning reach, comprehension, capacity to act, delivered protection, and health or safety benefit.

## 4 Critical comparison of contested system designs

### 4.1 Standardized vs. adjusted thresholds

Standardized categories support consistent communication, regulatory clarity, and a minimum protection floor (9, 15). Their weakness is limited sensitivity to task, sun, PPE, microclimate, and worker vulnerability. Adjusted thresholds improve occupational relevance and are supported by modeling, forecast translation, and physiological evidence (14, 17–19), but increasing complexity can reduce transparency, complicate supervision, and create dependence on proprietary algorithms. Representation is also uneven: WBGT-based research includes limited evidence for women and workers at the youngest and oldest ages used in standards (39). Current evidence does not establish universal superiority of either approach. A defensible interim model is a standardized minimum threshold supplemented by transparent, validated task adjustments and conservative escalation for vulnerable workers.

### 4.2 Advisory messages vs. mandatory organizational controls

Advisory alerts preserve flexibility and may be appropriate where workers and supervisors have genuine discretion. However, hydration or reduced-exertion advice is weak when schedules, production targets, staffing, and pay are controlled by employers or contractors. Field interventions show that individual behavior depends on available recovery arrangements and workload adjustment (23, 26–28). Policy evaluations in Italy and China suggest that stronger organizational duties can reduce injury outcomes under some conditions (30, 31), but direct comparisons with advisory protocols are scarce. The relevant question is therefore not simply voluntary vs. mandatory wording, but whether thresholds are clear, employers have capacity to comply, rest time is paid, retaliation is prevented, and enforcement reaches subcontracted and informal work.

### 4.3 Digital personalization vs. validation, privacy, and access

Apps, wearables, IoT platforms, and physiological models can make warnings timely and individualized (18–22). Their evidence base is dominated by technical performance, modeling, and usability rather than independently evaluated health outcomes. Sensors may be affected by placement, device performance, task conditions, and individual variation. Digital occupational-safety reviews identify surveillance, secondary data use, informed consent, unintended adverse effects, and weak data governance as recurring concerns (40). App-only systems may also exclude workers with limited connectivity, shared devices, low digital literacy, or restricted phone use.

Personal cooling technologies may complement alert-triggered controls but should not be confused with warning systems. For example, a 2026 numerical study of a phase-change-material textile cooling cap estimated prolonged thermal-comfort benefits under hot conditions (41). This expands the repertoire of possible responses, yet it does not evaluate warning generation, organizational implementation, or occupational health outcomes. Digital or personal technologies add value only when externally validated, used with informed consent and data minimization, made equitably accessible, and prevented from displacing collective protections or transferring responsibility from employers to workers.

## 5 Persistent evidence and implementation gaps

**Figure 1:**
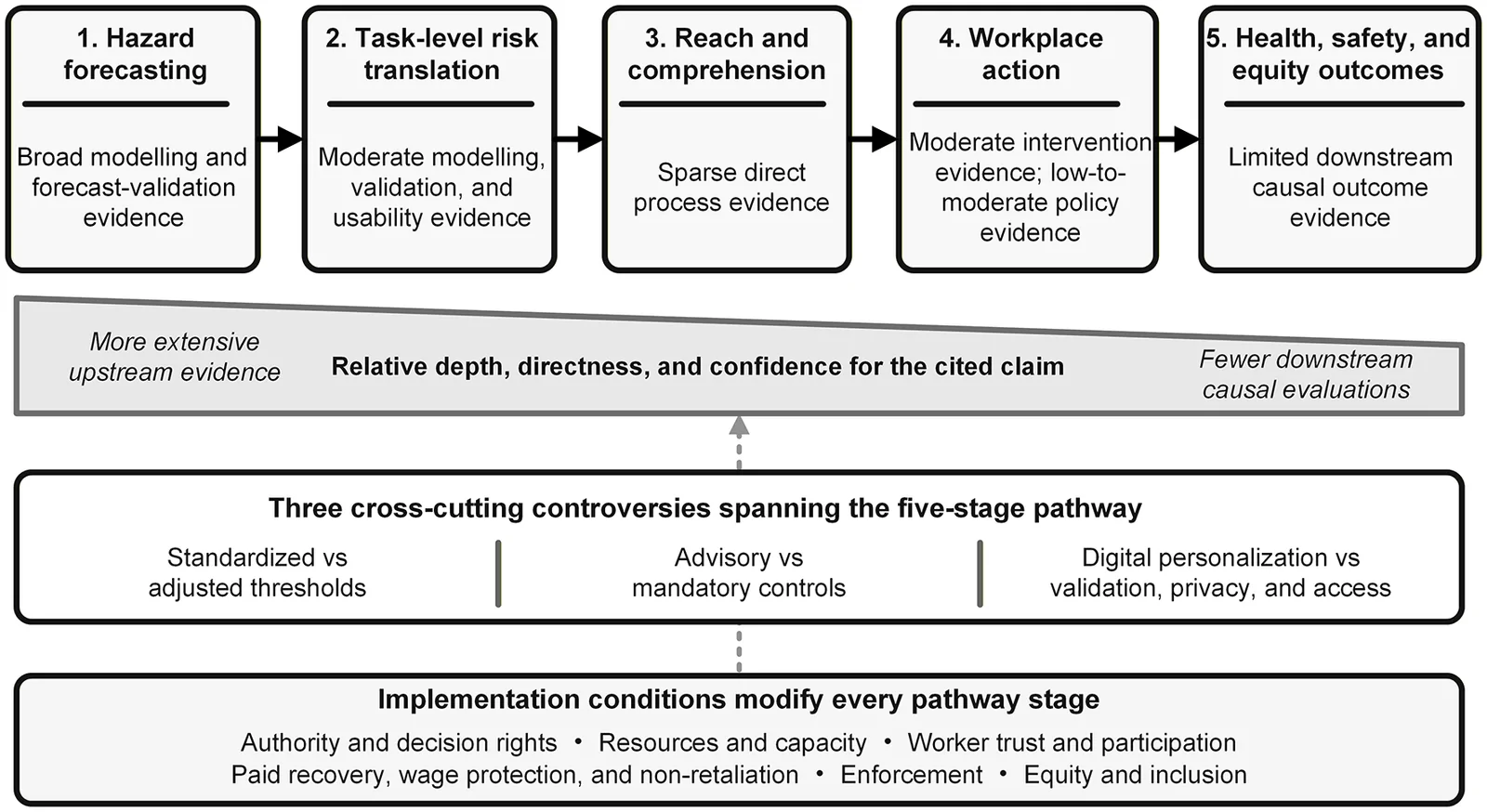
Evidence gradient across the occupational heat-warning pathway. The pathway extends from hazard forecasting through task-level risk translation, warning reach and comprehension, and workplace action to health, safety, and equity outcomes. Evidence descriptors summarize study design, occupational directness, outcome proximity, implementation reporting, and claim-specific confidence; they are qualitative judgments rather than formal certainty grades. The controversy and implementation bands span all five stages. Implementation conditions modify whether an alert produces protective action at each stage.

The evidence gradient is primarily a gradient in design and outcome proximity. Source-level appraisal indicated higher confidence for the broad occupational heat burden, supported by systematic reviews and meta-analyses (3–6), and moderate confidence that layered field action packages can reduce physiological strain or dehydration (26–29). Confidence in policy-linked injury reductions is low-to-moderate because the available evaluations are context-specific and vulnerable to residual confounding (30, 31). Confidence that digital or personalized warning tools reduce illness or injury remains low because most studies report modeling, technical performance, or usability rather than downstream outcomes (17–22). Figure 1 summarizes this qualitative gradient; Table 1 compares representative evidence, and Table 2 synthesizes the confidence assigned to key claims. Earlier systematic work on general HHWSs found heterogeneous designs and limited epidemiological evidence of effectiveness (42), while population studies linking extreme heat with hospitalizations can inform thresholds without showing whether workplace protocols were implemented (43).

**Table 1:** Comparative evidence on representative occupational heat-warning systems, component interventions, and policy-linked controls.

System or evidence	Setting and pathway component	Design and outcome	Main finding	Confidence for cited claim^*^	Main limitation or transferability
HEAT-SHIELD (12)	Europe; forecasting and task translation	Platform description and forecast framework	Links WBGT forecasts with workload, clothing, acclimatization, and lead times	Moderate for technical capability; low for downstream prevention	Limited direct evidence on workplace uptake or health outcomes
WORKLIMATE and risk-linked bans (13, 30)	Italy; forecasting, action, and injury outcome	Forecast modeling plus policy evaluation	Construction injuries declined during risk-linked restrictions; agricultural estimate was non-significant	Low-to-moderate for injury reduction	Policy, enforcement, and sector context may not transfer
California farmworker forecasts (14)	California agriculture; task translation	High-resolution modeling of WBGT-based rest-break recommendations	Demonstrates location-, shift-, season-, and acclimatization-specific translation	Moderate for task-level modeling	No direct measurement of implementation or health effects
ClimApp and wearable/IoT systems (19–22)	Thermal guidance and construction; personalized monitoring	Usability studies, scoping evidence, and technology validation	Supports individualized guidance and real-time early-warning feasibility	Moderate for feasibility; low for health benefit	Independent validation, privacy, access, and health-outcome evidence remain limited
Water-rest-shade and work redesign (26–28)	Sugarcane agriculture; workplace action	Field interventions with physiological, hydration, kidney, and productivity outcomes	Layered action packages reduced heat strain or dehydration	Moderate for physiological and hydration outcomes	Generally not forecast-triggered complete HHWSs
TEMP multifaceted programme (29)	Southern China power-grid workers; action and illness outcome	Cluster randomized trial; 528 workers	Illness incidence 1.80% vs. 4.82%; one follow-up showed significant adjusted reduction	Moderate for the programme effect	Multi-component design does not isolate the monitoring/warning component; later estimates imprecise
Guangzhou labor-protection policy (31)	China; mandatory control and injury outcome	Policy evaluation	Associated with lower heat-attributable injuries and insurance costs	Low-to-moderate for injury reduction	Residual confounding and uncertainty about enforcement and component effects
Construction spatial and physiological models (17, 18)	Construction; hazard characterization and task translation	Spatial and thermo-physiological modeling	Identifies hotspots, peak times, and worker-specific physiological risk	Moderate for risk characterization; low for health benefit	Does not establish comprehension, work modification, or health benefit
PCM-textile cooling cap (41)	Hot-climate personal cooling; response component	Numerical thermal-performance study	Illustrates a possible personal cooling measure complementing action plans	Low and indirect for occupational protection	Not a warning system; no organizational implementation or occupational health evaluation

^*^Confidence applies only to the claim for which the source is cited and is a narrative judgment, not a formal GRADE rating.

**Table 2:** Key claim synthesis and claim-specific confidence.

Key claim	Claim-specific confidence	Principal evidence	Main reason for rating	Supported interpretation
Occupational heat exposure is associated with adverse health, physiological, productivity, and injury outcomes	Higher	Systematic reviews/meta-analyses and recent scoping evidence (3–6)	Consistent direct occupational evidence, although heterogeneous across jobs and climates	The evidence supports an association, while effect sizes remain context-dependent.
Occupationally relevant forecasts and task-adjusted translation are technically feasible	Moderate	HEAT-SHIELD, WORKLIMATE, California farmworker modeling, and construction models (12–14, 17, 18)	Direct occupational modeling/validation, but little downstream implementation evidence	The evidence supports technical feasibility and decision-support capability; downstream health effectiveness remains unestablished.
Layered workplace action packages can reduce heat strain or dehydration	Moderate	Sugarcane field interventions (26–28)	Direct worker outcomes but largely non-randomized and concentrated in one sector	The evidence supports possible reductions in heat strain or dehydration within the studied sectors; generalizability remains limited.
A multifaceted occupational heat programme can reduce heat-related illness	Moderate	Cluster randomized TEMP trial (29)	Randomized clusters and illness outcome; multi-component attribution and imprecision remain	The observed effect applies to the multifaceted programme; the independent contribution of monitoring or alerts cannot be isolated.
Policy-linked restrictions or labor protections may reduce occupational injuries	Low-to-moderate	Italy and Guangzhou policy evaluations (30, 31)	Relevant injury outcomes but residual confounding, enforcement variation, and limited transferability	The evidence supports association-level rather than causal interpretation.
Digital personalization, wearables, or IoT warning tools reduce illness or injury	Low	ClimApp, wearable review, IoT system, and physiological models (18–22)	Predominantly modeling, technical performance, and usability evidence	Independent evidence of illness or injury reduction is not yet available.
Occupational HHWSs distribute protection equitably across worker groups	Low	Migrant-worker, farmworker, regional, and digital-access evidence (32–37, 40)	Mostly descriptive evidence and sparse comparative outcome measurement	Equity is supported primarily as an implementation concern and evidence gap rather than an established outcome.

The implementation pathway remains poorly observed. Studies rarely report who received an alert, how quickly work changed, whether recovery periods occurred as prescribed, whether supervisors complied, or whether planned resources were available (23, 30, 32). This prevents separation of forecast failure from communication failure or implementation failure. Evaluation should pre-specify measures for forecast performance, reach, comprehension, implementation fidelity, work modification, and health, injury, productivity, cost, and equity outcomes. Reporting only forecast skill cannot demonstrate prevention, while reporting only a final outcome can conceal the stage at which the pathway failed.

Comparative detail is also uneven across sectors and enforcement settings. Agriculture and construction dominate the evidence, while mobile crews, utilities, waste work, emergency response, small enterprises, and informal work remain underrepresented. A technically effective alert may widen inequity if only well-resourced employers can act. Table 3 presents an equity-failure matrix linking underserved groups to the pathway stage at which protection may fail, the likely mechanism, and the system feature needed to address it. Research should test complete warning-and-action protocols through natural experiments, controlled before-after studies, interrupted time-series analyses, matched worksites, or prospectively staged implementation.

**Table 3:** Equity failure matrix across the occupational heat-warning pathway.

Underserved group/condition	Likely pathway failure stage	Failure mechanism	System design or implementation response	Illustrative evidence
Migrant and linguistic-minority workers	Reach and comprehension	Language mismatch, low trust, unfamiliar terminology, limited access to occupational health communication	Multilingual redundant channels, trusted intermediaries, worker testing of message comprehension	32–35
Piece-rate, temporary, and insecurely employed workers	Workplace action	Income loss, retaliation risk, and limited discretion to reduce pace or stop work	Paid recovery time, non-retaliation protection, supervisor accountability, wage protection	32–34
Subcontracted workers and multi-employer sites	Decision authority and implementation fidelity	Fragmented responsibility between principal contractors, subcontractors, supervisors, and occupational health staff	Named duty holder, documented escalation route, cross-employer compliance audit	23, 32
Women, age extremes, new and unacclimatized workers	Threshold validity and task translation	Underrepresentation in standards and physiological evidence; variable vulnerability	Conservative escalation and validated subgroup/task adjustments	15, 39
Workers in heavy tasks or impermeable PPE	Task-level risk translation	Regional or standard WBGT may underrepresent metabolic and clothing burden	On-site measurement, workload/PPE adjustment, shorter reassessment interval	1, 14–16
Small enterprises, informal workers, and mobile crews	Implementation capacity	Limited staffing, sensors, occupational-health expertise, shade, transport, and enforcement coverage	Low-cost fallback protocols, shared services, mobile monitoring, regulator support	32, 36, 37
Workers with limited connectivity, shared devices, or restricted phone use	Communication and digital access	App-only delivery excludes workers or creates inconsistent access	Non-digital backup channels, shared display, supervisor relay with confirmation	21, 22, 40
Workers concerned about surveillance or punitive data use	Digital personalization and trust	Physiological data may be repurposed for productivity management or discrimination	Informed consent, data minimization, access controls, retention limits, non-punitive governance	22, 40

### 5.1 Future research and practice priorities

The next phase of research should compare complete warning-and-action protocols rather than isolated technical components. Policy changes and phased employer implementation create opportunities for natural experiments, controlled before-after comparisons, interrupted time-series analyses, and matched worksite studies; cluster or stepped-wedge designs may be feasible where implementation can be staged prospectively. These designs could compare fixed vs. task-adjusted thresholds, voluntary vs. mandatory measures, and app-only vs. supervisor-integrated communication while measuring unintended operational effects.

Different designs answer different questions. Sensor and forecast validation establish measurement accuracy; process evaluation explains reach, comprehension, adoption, and fidelity; randomized and quasi-experimental evaluations estimate intervention effects; and economic evaluation tests whether avoided illness, injury, absenteeism, turnover, and productivity loss justify implementation costs. The power-grid trial illustrates the value of measuring health outcomes and uptake while showing why multi-component programmes need component-specific process evaluation (29). Studies should report negative and null findings, false alarms, non-compliance, and sectors in which controls were infeasible.

Future research must broaden geographic and employment coverage. Locally led studies should test feasible action packages in low-resource settings, informal work, small enterprises, and dispersed or mobile occupations. Analyses should examine differences by sex and gender where relevant, age, acclimatization, migration status, contract type, work intensity, PPE, and climate zone. Paid recovery time, non-retaliation protection, replacement staffing, procurement capacity, and enforcement should be measured as implementation conditions rather than assumed.

## 6 Discussion

This review differs from recent syntheses of occupational heat hazards and preventive recommendations by organizing evidence across the full warning-to-action pathway and distinguishing complete systems from component studies. Table 4 contrasts this contribution with recent reviews of population HHWSs, heat-action plans, occupational heat burden, and outdoor-worker adaptation. High-resolution WBGT forecasts, physiological models, and IoT tools demonstrate increasing technical capability, but most do not show that warnings were understood, that supervisors modified work, or that illness and injury declined. Conversely, field interventions, the power-grid trial, and labor policies provide more direct action or outcome evidence, yet may not identify which forecast, threshold, communication, or enforcement component produced the effect.

**Table 4:** Comparison with recent reviews of heat-health warning systems, occupational heat, and outdoor-worker adaptation.

References	Primary scope	Typical evidence emphasis	Limitation relative to current question	Relationship to the present review
Chandra and Lee (9)	Heat-health warning systems, mainly population-level	Frameworks, thresholds, implementation variation, health outcomes	Limited occupational task and employer-action focus	Extends population-level HHWS evidence to occupational task translation and workplace implementation.
Kotharkar and Ghosh (10)	Heat-health action plans, 1995–2020	Plan components and management approaches	Predates much 2024–2026 occupational digital and policy evidence	Adds recent occupational evidence, claim-specific appraisal, and downstream action gaps.
Bhati and Sheth (11)	Global occupational heat stress, adaptation, and policy	Broad hazards, adaptation, and policy responses	Does not center the complete alert-to-action mechanism	Separates complete occupational HHWSs from component interventions.
Habibi et al. (24)	Heat-resilient outdoor workers	Risk factors and control strategies	Broad prevention focus rather than warning-system pathway	Links forecasts with workplace decisions, implementation, and outcomes.
Edgerly et al. (23)	Prevention recommendations for outdoor workers	Preparation, monitoring, and response recommendations	Recommendations are not comparative effectiveness evidence	Distinguishes recommendations from intervention, policy, and outcome evidence.
Present review	Occupational HHWSs for outdoor workers	Forecasting, task translation, reach, action, outcomes, controversies, implementation and equity	Structured narrative design without exhaustive database coverage or a PRISMA flow	Integrates complete-system and component evidence across the warning-to-action pathway, with claim-specific confidence judgments and a structured equity analysis.

The available evidence therefore supports conditional rather than universal conclusions. Occupationally adjusted thresholds are more job-relevant, but require transparent derivation and validation. Mandatory controls may overcome limited worker discretion, but their effects depend on enforcement, wage protection, and employer capacity. Digital personalization may identify changing risk, but its benefit is uncertain when data governance, external validation, or equitable access is weak. These are not merely technical trade-offs; they determine who has authority to act and who bears the consequences of false reassurance, false alarms, or production disruption.

The review has limitations. It used a structured, purposive narrative approach rather than a prospectively registered systematic-review design. Searches prioritized recent English-language literature and selected foundational sources; relevant local policy documents or non-English evaluations may have been missed. Confidence judgments remain narrative rather than formal GRADE ratings, and several conclusions rely on component interventions, modeling, or policy associations.

These limitations also define a research agenda. Future studies should compare complete protocols across sectors and climates, state the decision supported by each data layer, report implementation fidelity and enforcement conditions, and examine distribution of protection across employment groups. Co-design with workers, employers, occupational health practitioners, worker organizations, meteorological services, and regulators can identify barriers before deployment. A common minimum reporting set would improve transferability by documenting the forecast source, lead time, threshold derivation, task adjustments, communication channel, responsible decision-maker, prescribed and actual controls, deviations, and outcomes.

## 7 Conclusions

Occupational HHWSs are advancing technically faster than they are being evaluated as complete preventive systems. Confidence is strongest for the occupational heat burden and for selected field action packages, but remains limited for downstream effects of digital or personalized warning technologies and only low-to-moderate for policy-linked injury reductions. Effective protection requires credible forecasts, transparent task-level decisions, timely communication, enforceable workplace actions, and equitable capacity to comply. Comparative studies should follow the full warning-to-action pathway and identify where systems fail, for whom, and under which organizational and regulatory conditions.
